# Effects of overexpression of ephrin-B2 on tumour growth in human colorectal cancer

**DOI:** 10.1038/sj.bjc.6601723

**Published:** 2004-03-23

**Authors:** W Liu, Y D Jung, S A Ahmad, M F McCarty, O Stoeltzing, N Reinmuth, F Fan, L M Ellis

**Affiliations:** 1 Department of Cancer Biology, The University of Texas M.D. Anderson Cancer Center, 1515 Holcombe Boulevard, Houston, TX 77030-4009, USA; 2 Department of Surgical Oncology, The University of Texas M.D. Anderson Cancer Center, Unit 444, 1515 Holcombe Boulevard, Houston, TX 77030-4009, USA

**Keywords:** ephrin-B2, colorectal cancer, angiogenesis, tyrosine kinase, receptor, ligand

## Abstract

Eph receptor tyrosine kinases (RTKs) and their membrane-bound ligands, the ephrins, are essential for embryonic vascular development. Recently, it has been demonstrated that overexpression of specific Ephs and ephrins is associated with a poor prognosis in human tumours. Our group has shown that EphB and the ephrin-B subfamilies are coexpressed in human colorectal cancer, and ephrin-B2 is expressed at higher levels in human colorectal cancer than in adjacent normal mucosa. As the Eph/ephrin system is involved in embryologic vasculogenesis and ephrin-B2 is expressed ubiquitously in all colon cancers studied in our laboratory, we hypothesised that overexpression of ephrin-B2 in colon cancer cells may induce tumour angiogenesis and increase tumour growth. To investigate this hypothesis, we stably transfected KM12L4 human colon cancer cells with ephrin-B2 to study its effect on tumour growth *in vivo*. We found that overexpression of ephrin-B2 markedly decreased tumour growth in a mouse xenograft model. Immunohistochemical staining showed that ephrin-B2 transfectants produced higher tumour microvessel density and lower tumour cell proliferation than did parental or vector-transfected control cells. Using ^51^Cr-labelled red blood cells (RBCs) to determine the functional blood volume in tumours, we demonstrated that tumours from ephrin-B2-transfected cells had significantly decreased blood volume compared with tumours from parental or vector-transfected control cells. Evaluation of *in vitro* parameters of cell cycle mediators demonstrated no alteration in the cell cycle. Although ephrin-B2 transfection increased tumour vessel density, the decrease in blood perfusion suggests that these vessels may be ‘dysfunctional’. We conclude that overexpression of ephrin-B2 suppresses tumour cell growth and vascular function in this *in vivo* colon cancer model.

The largest subfamily of receptor tyrosine kinases (RTKs), the Eph family, and their ligands (ephrins), play important roles in physiologic and pathologic vascular processes. Both the Ephs (receptors) and ephrins (ligands) are divided into two groups, A and B subfamilies (Eph Nomenclature Committee, 1997). The binding of ephrin ligands to Eph receptors is dependent on cell–cell interactions. The interactions of ephrins and Ephs have recently been shown to function via bi-directional signalling. The ephrins binding to Eph receptors initiate phosphorylation at specific tyrosine residues in the cytoplasmic domain of the Eph receptors. In response to Eph receptor binding, the ephrin ligand also undergoes tyrosine phosphorylation, so-called ‘reverse’ signalling (Holland *et al*, 1996; Bruckner *et al*, 1997).

Eph RTKs and their ephrin ligands play important roles in embryonic vascular development. Disruption of specific Eph receptors and ligands (including ephrin-B2) leads to defective vessel remodelling, organisation, and sprouting resulting in embryonic death (Wang *et al*, 1998; Adams *et al*, 1999; Gale and Yancopoulos, 1999; Helbling *et al*, 2000). Coordinated expression of the Eph/ephrin system determines the phenotype of embryonic vascular structures: ephrin-B2 is present on arterial endothelial cells (ECs), whereas EphB4 is present on venous ECs (Gale and Yancopoulos, 1999; Shin *et al*, 2001). Recently, specific Ephs and ephrins have been implicated in tumour growth and angiogenesis. The Ephs and ephrins have been found to be overexpressed in many human tumours (Kiyokawa *et al*, 1994; Vogt *et al*, 1998; Bruce *et al*, 1999; Tang *et al*, 1999a, 1999b; Walker-Daniels *et al*, 1999; Ogawa *et al*, 2000; Varelias *et al*, 2002), and higher expression levels of Ephs and ephrins have been found to correlate with more aggressive and metastatic tumours (Vogt *et al*, 1998; Nakamoto and Bergemann, 2002).

Our group has shown that EphB and the ephrin-B subfamilies are co-expressed in human colorectal cancer, and ephrin-B2 is expressed at higher levels in human colorectal cancer than in adjacent normal mucosa (Liu *et al*, 2002). As the Eph/ephrin system is involved in embryologic vasculogenesis and ephrin-B2 is expressed ubiquitously in all colon cancers studied in our laboratory, we hypothesised that overexpression of ephrin-B2 in colon cancer cells may induce tumour angiogenesis and increase tumour growth. We found that overexpression of ephrin-B2 in colon cancer cells did lead to an increase in tumour angiogenesis, but that these vessels were not functional. Paradoxically, overexpression of ephrin-B2 was associated with a marked decrease in tumour growth that was likely secondary to the development of an inefficient vascular network.

## MATERIALS AND METHODS

### Cells and cell cultures

The KM12L4, KM20, KM23, KM12C, and KM12SM human colon cancer cell lines were provided by Dr IJ Fidler (The University of Texas M.D. Anderson Cancer Center, Houston, TX, USA) (Morikawa *et al*, 1988). HT-29 (human colorectal cancer cells), MRC-5 (human lung fibroblasts), 10T1/2 (murine fibroblasts), CCD-33Co and CCD-112Co (human colorectal fibroblasts), and human umbilical vein endothelial cells (HUVECs) were purchased from the American Type Culture Collection (ATCC, Manassas, VA, USA). MRC-5, CCD-33Co, CCD-112Co, and all colon cancer cell lines were cultured in minimal essential medium (MEM) supplemented with 10% foetal bovine serum (FBS), penicillin–streptomycin, vitamins, sodium pyruvate, L-glutamine, and nonessential amino acids (Life Technologies, Inc., Grand Island, NY, USA). Murine 10T1/2 cells were grown in basal Eagle's medium supplemented with 10% FBS, and 2 U ml^−1^ penicillin–streptomycin. HUVECs were cultured on 0.5% gelatin (Sigma Chemical Co. St Louis, MO, USA) in modified MEM supplemented with 15% FBS, 10 ng ml^−1^ recombinant human basic fibroblast growth factor, penicillin–streptomycin, vitamins, sodium pyruvate, L-glutamine, and nonessential amino acids. All cells were grown at 37°C in 5% CO_2_ and 95% air.

### Antibodies for immunohistochemistry

Antibodies for immunohistochemical analysis were obtained as follows: rabbit anti-human ephrin-B2 antibody (Santa Cruz Biotechnology, Santa Cruz, CA, USA), rat anti-mouse CD31/PECAM-1 antibody and peroxidase-conjugated rat anti-mouse immunoglobulin G1 (IgG1) from Pharmingen (San Diego, CA, USA); mouse antiproliferating cell nuclear antigen (PCNA) clone PC10 DAKO A/S from DAKO Corp. (Carpinteria, CA, USA); and peroxidase-conjugated goat anti-rat IgG (H+L) and peroxidase-conjugated rat anti-mouse IgG2a from Serotec Harlan Bioproducts for Science, Inc. (Indianapolis, IN, USA).

### Isolation of mRNA and Northern blot analysis

Polyadenylated mRNA was extracted from 10^7^–10^8^ cells from colon cancer cell lines or nonmalignant cell lines growing in culture by using a FastTrack mRNA isolation kit (Invitrogen Corp., San Diego, CA, USA). Northern blot analysis was performed as previously described (Ellis *et al*, 1996). After prehybridisation, the membranes were probed for ephrin-B2 full-length cDNA (the probe was a generous gift from Dr Renping Zhou, Laboratory for Cancer Research, College of Pharmacy, Rutgers University, Piscataway, NJ, USA; Yue *et al*, 1999), EphB4 (the probe was a purified polymerase chain reaction product with the primer sequences as follows: forward, 5′-GTCTGACTTTGGCCTTTCCC-3′; reverse, 5′-TGACATCACCTCCCACATCA-3′), and glyceraldehyde-phosphate dehydrogenase (internal control; ATCC). Each cDNA probe was purified by agarose gel electrophoresis, recovered by using a QIAEX gel extraction kit (QIAGEN Inc., Chatsworth, CA, USA), and radiolabelled by a random primer technique with a commercially available kit (Amersham Life Science Inc., Arlington Heights, IL, USA). Nylon filters were washed at 65°C with 30 mmol l^−1^ NaCl, 3 mmol l^−1^ sodium citrate (pH 7.2), and 0.1% sodium dodecyl sulphate. Autoradiography was then performed.

### Subcloning of ephrin-B2 into pcDNA3 and DNA transfection

Ephrin-B2 was subcloned into the Kpn I/Not I sites of pcDNA3 (Invitrogen), a eukaryotic expression vector driven by the human cytomegalovirus promoter and containing a neomycin-resistance gene. Completeness and orientation of the insert were determined by means of restriction enzyme analyses and DNA sequencing (Core Sequencing Facilities, M.D. Anderson Cancer Center).

The ephrin-B2 sense plasmid as well as vector plasmid alone (pcDNA3) were transfected into KM12L4 cells utilising the FuGENE 6 transfection system, according to the manufacturer's protocol (Boehringer Mannheim Co., Indianapolis, IN, USA). At 48 h after transfection, medium containing G418 (400 *μ*g ml^−1^) was used to select for clones. Individual colonies were expanded and examined for ephrin-B2 expression by Northern blot analysis. Clones with a high expression of exogenous ephrin-B2 were pooled to establish an additional experimental cell line, the ephrin-B2 S pool.

### Animals and tumour cell inoculation

Male nude mice (8 weeks old) were obtained from the National Cancer Institute's Animal Production Area (Frederick, MD, USA), acclimated for 1 week, caged in groups of five, and fed a diet of animal chow and water *ad libitum* throughout the experiment. Mice were randomised to one of the four groups, with similar mean body weights among the four groups. With a 30-gauge needle and a 1-ml syringe, 200 *μ*l (containing 1 × 10^6^ cells) of KM12L4, pcDNA3-transfected KM12L4, or ephrin-B2-sense transfected KM12L4 (S2 or S pool) cells were injected subcutaneously in the right upper flank of 10 mice each. Prior to injection, a trypan blue exclusion test was carried out to assure that cell viability was greater than 80%. Tumour growth was measured every other day. Tumour volume was calculated using the formula: volume=(diameter^2^ × length)/2. All animal studies were conducted according to a protocol approved by the Institutional Animal Care and Use Committee (IACUC) of M.D. Anderson Cancer Center and were performed in accordance with the UKCCCR guidelines for the welfare of animals in experimental neoplasia (Workman *et al*, 1998). All animals were euthanised 15 days after tumour cell inoculation because of the large volume of the KM12L4 tumours (maximum size permitted by the IACUC), and the tumours were harvested for further analysis. Harvested tumours were placed in 10% formalin for paraffin fixation or placed in optimum cutting temperature (OCT; Miles Inc., Elkhart, IN, USA) solution and snap frozen for frozen sectioning. This experiment was performed three times with similar results.

### Immunohistochemistry of paraffin-embedded and frozen tissues and immunofluorescent staining of cells

Paraffin-embedded tumours were sectioned (4–6 *μ*m thick), mounted on positively charged Superfrost slides (Fisher Scientific Co., Houston, TX, USA), and allowed to dry overnight at room temperature. Sections were deparaffinised in xylene followed by 100, 95, and 80% ethanol and rehydrated in phosphate-buffered saline (PBS; pH 7.5). These sections were used for haematoxylin and eosin (H&E) staining and detection of PCNA. Sections analysed for PCNA were microwaved for 5 min to increase antigen retrieval before staining, and stained as described below.

Tumours frozen in OCT solution were sectioned (8–10 *μ*m thick), mounted on positively charged Superfrost slides, and air-dried for 30 min. Tissues were fixed in cold acetone (5 min), 1 : 1 acetone/chloroform (5 min), and acetone (5 min) and then washed with PBS three times for 3 min each. These sections were used for CD31 staining. Specimens were then incubated with 3% hydrogen peroxide in PBS (v v^−1^) for 12 min at room temperature to block endogenous peroxidase. Sections were washed three times for 3 min each with PBS (pH 7.5) and incubated for 20 min at room temperature in a protein-blocking solution consisting of PBS supplemented with 1% normal goat serum and 5% normal horse serum.

The primary antibodies directed against CD31 and PCNA were diluted 1 : 100 and 1 : 50, respectively, in protein-blocking solution and applied to the sections, which were then incubated overnight at 4°C. Sections were then rinsed three times for 3 min each in PBS and incubated for 10 min in protein-blocking solution before the addition of peroxidase-conjugated secondary antibody. The secondary antibodies used to stain for CD31 [peroxidase-conjugated goat anti-rat IgG (H+L)] and PCNA (peroxidase-conjugated rat anti-mouse IgG2a) were diluted 1 : 200 and 1 : 100, respectively, in protein-blocking solution. After incubation with the secondary antibody for 1 h at room temperature, the samples were washed and incubated with stable diaminobenzidine (Research Genetics, Huntsville, AL, USA) substrate. Staining was monitored under a bright field microscope, and the reaction was stopped by washing with distilled water. For the CD31 staining, sections were counterstained with Gill's No. 3 haematoxylin (Sigma Chemical Co.) for 15 s and mounted with Universal Mount (Research Genetics). Control specimens were treated with a similar procedure except that the primary antibody was omitted.

Ephrin-B2-sense-transfected cells (S2) and vector-transfected cells (KM12L4/pcDNA3) were seeded in the wells of four-chamber slides (Fisher Scientific Co.) and cells were grown until 80% confluent. The cells were fixed with cold acetone (Sigma Chemical Co.) for 10 min and washed with PBS twice for 5 min each. Immunofluorescent staining was performed as previously described (Liu *et al*, 2002) with the following modifications. After the cells were incubated overnight at 4°C with the primary antibody (ephrin-B2, 1 : 100 dilution), washed, and incubated with protein-blocking solution, they were incubated with the Alexa 594-conjugated secondary antibody (1 : 400 dilution of Alexa 594 goat anti-rabbit IgG (H+L); (Molecular Probes, Eugene, OR, USA)) for 60 min at room temperature. The slides were then washed with PBS and the coverslips were mounted on glass slides using mounting solution consisting of Hoechst 33342 fluorescent dye (Sigma Chemical Co.) and PBS.

### Quantification of tumour microvessel density and tumour cell proliferation

To quantify tumour microvessel density and tumour cell proliferation, tumour microvessels (stained for CD31) and PCNA-positive cells were evaluated by light microscopy and counted in three random 0.159 mm^2^ fields at × 100 magnification using a Sony 3-chip camera (Sony, Montvale, NJ, USA) mounted on a Zeiss universal microscope (Carl Zeiss, Thornwood, NY, USA) and Optimas Image Analysis software (Bioscan, Edmond, WA, USA) installed on a Compaq computer with Pentium chip, a frame grabber, an optical disk storage system, and a Sony Mavigraph UP-D7000 digital color printer (Tokyo, Japan). Microvessels were quantified according to the method described by Ahmad *et al* (2001).

### Determination of functional blood volume/flow in tumours

Five nude mice were killed and their blood was harvested in heparinised syringes (approximately 1 ml per mouse) for radiolabelling. Red blood cells (RBCs) were centrifuged at 1000 rpm for 5 min and resuspended in 1 ml of PBS containing Hank's balanced salt solution (HBSS) (Life Technologies). In all, 1 mCi of radiochromium (^51^Cr) (Amersham) was added to label the RBCs. The labelling reaction was carried out in a 37°C water bath with shaking for 2 h. The ^51^Cr-labelled RBCs were then washed twice with PBS for 15 min each time and resuspended in HBSS. A 0.2-ml suspension of ^51^Cr-labelled RBCs was injected intravenously into the tail vein of tumour-bearing mice ((*n*=10/group), ∼0.05 mCi per mouse). At 10 min after injection of ^51^Cr-labelled RBCs, 10 *μ*l of blood was collected from the tail vein of each mouse for radioactive count reading. The mice were then killed, the tumours were removed, and radioactive counts of the tumours were recorded with the Packard Auto-Gamma counter (Downers Grove, IL, USA). The blood volume in the tumours was calculated using the formula: blood volume (*μ*l g^−1^)=tumour radioactive count (cpm g^−1^)/blood radioactive count (cpm *μ*l^−1^).

### Statistical analysis

Tumour weights, tumour volumes, tumour microvessel densities, and numbers of PCNA-positive cells were compared between groups by using one-way analysis of variance (ANOVA). Functional blood volume in tumours (^51^Cr labelling) was compared using the Mann–Whitney *U* test for nonparametric data. All analyses were carried out using InStat Statistical Software (GraphPad Software, San Diego, CA, USA) with *P*⩽0.05 considered to be significant.

## RESULTS

### Ephrin-B2 and EphB4 mRNA expression in normal and colon cancer cell lines

**Figure 1:**
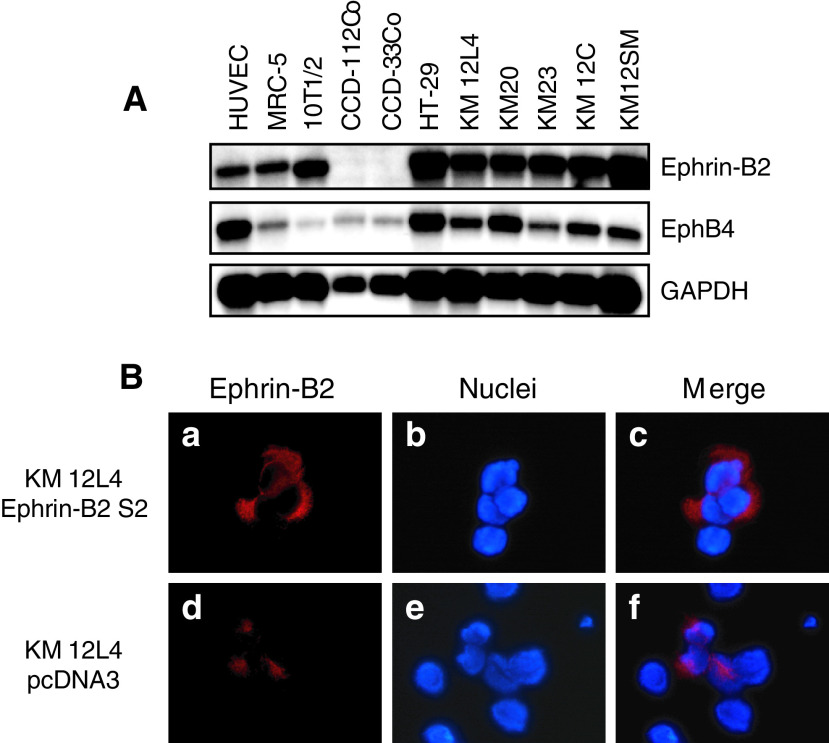
Expression of ephrin-B2 and EphB4 in cell lines and immunofluorescent staining of Ephrin-B2 in transfected colon cancer cell lines. (**A**) Coexpression of ephrin-B2 and EphB4 mRNA in the various normal and colon cancer cell lines. GAPDH, glyceraldehyde-phosphate dehydrogenase. (**B**) Immunofluorescent staining with ephrin-B2 was performed on ephrin-B2 transfected (S2) and vector transfected cells. Representative pictures showed that the staining of ephrin-B2 was much stronger (a, red) on the cell surface (c, merged image) in ephrin-B2-sense-transfected KM12L4 cells compared to the pcDNA3-transfected cells (d, red and f, merged image).

Northern blotting was performed to determine the frequency of expression of ephrin-B2 and EphB4 in human nonmalignant cell lines and colon cancer cell lines. All the nonmalignant cell lines and colon cancer cell lines examined expressed ephrin-B2 and EphB4 (Figure 1A

). However, the expression of EphB4 mRNA was lower in the nonmalignant cell lines, with the exception of HUVECs, than in the colon cancer cell lines.

### Transfection of Ephrin-B2 in to colon cancer cells and its effect on tumour growth

**Figure 2:**
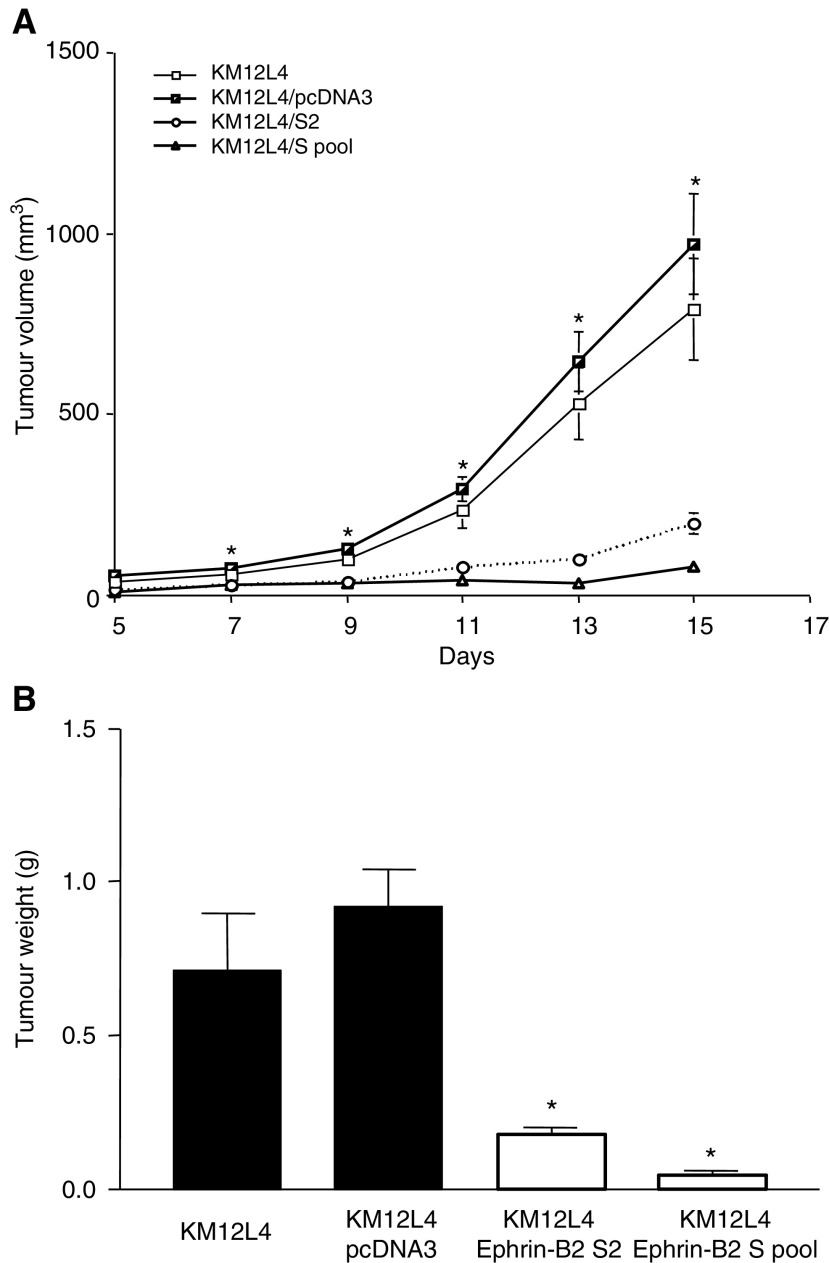
Effect of ephrin-B2 overexpression on the growth of tumours from ephrin-B2-sense-transfected KM12L4 cells in nude mice. (**A**) Mean tumour volumes were determined every other day following subcutaneous injection (*n*=10). On days 7–15, the mean volumes of tumours from ephrin-B2-sense-transfected cells (S2 and S pool) were significantly smaller than those of control tumours from parental KM12L4 cells or vector-transfected cells (^*^P<0.0001, ANOVA). Error bars indicate the standard error (s.e.m.) of the mean. (**B**) After 15 days, the mice injected with ephrin-B2-sense-transfected KM12L4 cells (white bars) had significantly less heavy tumours than did control mice (black bars) (^*^P<0.0001, ANOVA). Error bars indicate s.e.m. of the mean.

Northern blot analysis demonstrated higher levels of ephrin-B2 in transfected cells (S2 and S pool), as expected (data not shown). In addition, to confirm that the transfected ephrin-B2 protein product localised to the cell surface, we performed immunofluorescent staining on cells *in vitro*. In ephrin-B2 transfected cells, we noted localisation of the protein to the cell surface and its expression was greater than that in control cells (Figure 1B). As expected, the ephrin-B2 receptor, EphB4, was unchanged in the ephrin-B2-sense transfected cells (data not shown). To determine whether overexpression of ephrin-B2 affects the growth of KM12L4 tumours, we injected KM12L4 tumour cells transfected with a sense construct for ephrin-B2 subcutaneously in nude mice. Tumours from ephrin-B2-sense-transfected cells (S2 and S pool) had significantly smaller volumes (Figure 2A

) and weights (Figure 2B) than did tumours from parental KM12L4 or pcDNA3-transfected cells (controls). All mice were killed on day 15 since the tumours in the control groups were of maximal size as outlined by the IACUC. These findings were confirmed in two subsequent studies.

### Effect of ephrin-B2 overexpression on vessel density and tumour cell proliferation

**Figure 3:**
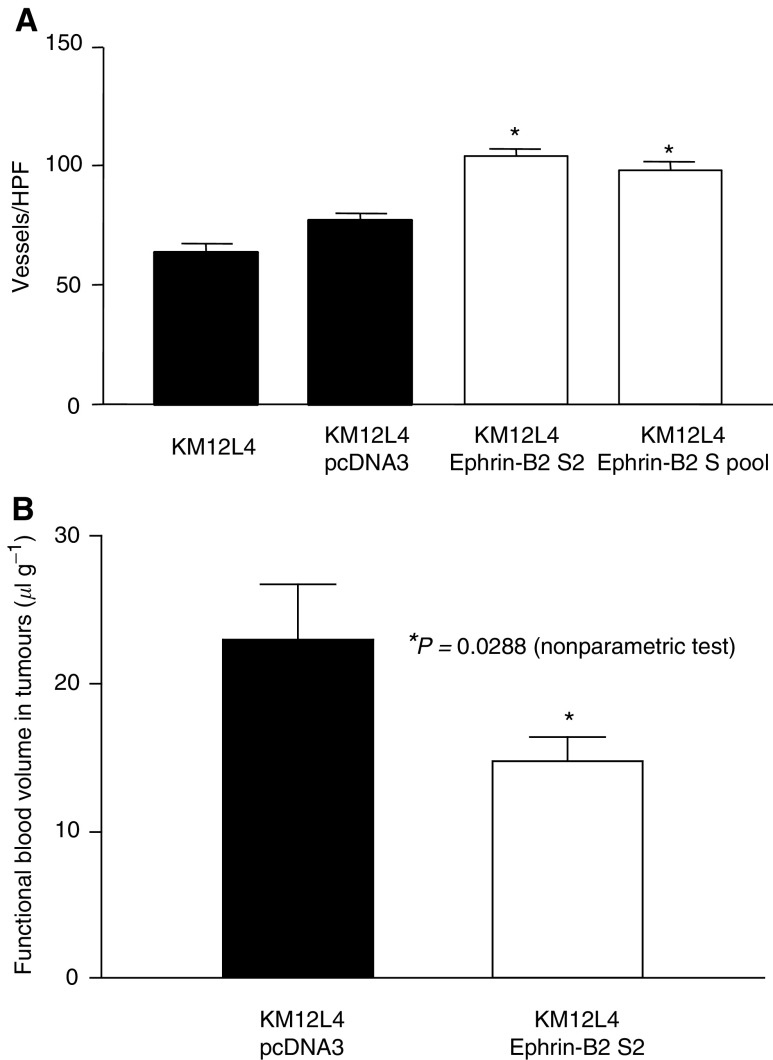
Effect of ephrin-B2 overexpression on tumour vessel counts and blood volume in tumours. (**A**) Immunohistochemical staining for CD31 in tumour sections was performed. The mean vessel count in tumours from mice injected with ephrin-B2-sense-transfected KM12L4 cells (white bars) was significantly greater than in control tumours (black bars) (^*^P<0.0001, ANOVA). Error bars indicate s.e.m. of the mean. HPF, high-power field. (**B**) ^51^Cr-labelled RBCs suspended in HBSS were injected into the tail vein of tumour-bearing nude mice. The mean blood volume in tumours from ephrin-B2-sense-transfected KM12L4 cells (S2) was significantly less than in control tumours from vector-transfected cells (^*^P=0.0288, nonparametric test). Error bars indicate s.e.m. of the mean.

**Figure 4:**
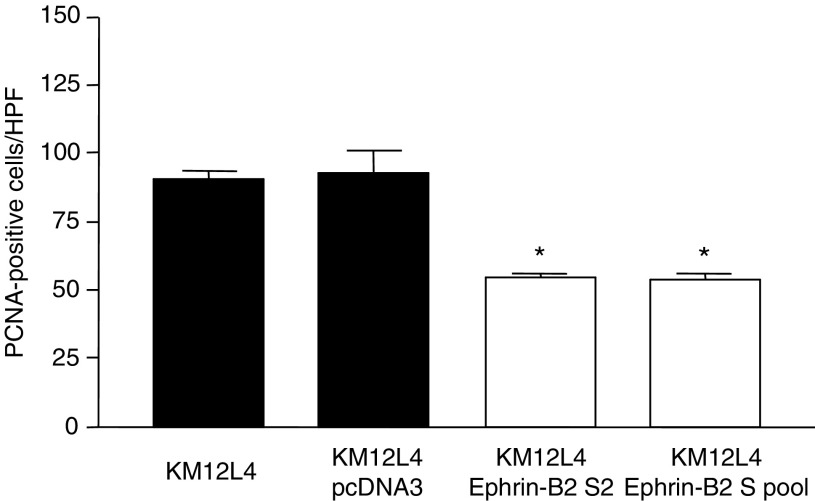
Effect of ephrin-B2 sense transfection on tumour cell proliferation. Immunohistochemical staining for PCNA in tumour sections was performed. The mean number of PCNA-positive cells in tumours from mice injected with ephrin-B2-sense-transfected KM12L4 cells (white bars) were significantly lower than in control tumours (black bars) (^*^P<0.0001, ANOVA). Error bars indicate s.e.m. of the mean. HPF, high-power field.

**Figure 5:**
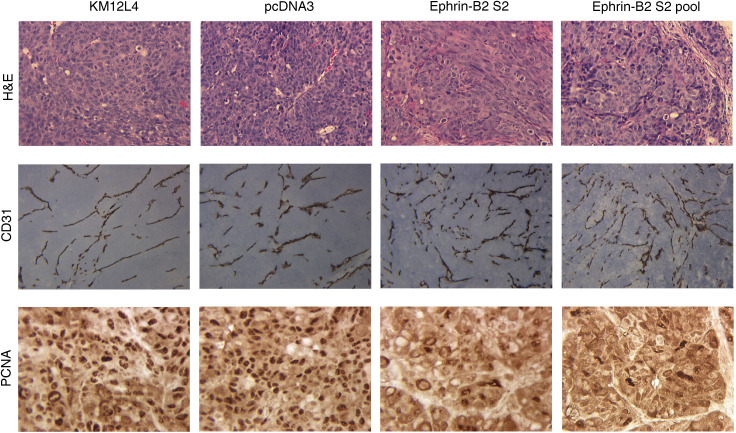
Immunohistochemical staining H&E, CD31, and PCNA. Immunohistochemical staining with H&E (top row; × 100), CD31 (middle row; × 100), and PCNA (bottom row; × 100) was performed on tumour sections. Representative sections demonstrate more vessels and fewer PCNA positive cells in tumours from ephrin-B2-sense-transfected KM12L4 cells (columns 3 and 4). The vessels in the experimental tumours were morphologically distinct from normal vessels in that they were small and less prominent that those in control tumours (columns 1 and 2).

Immunohistochemical staining for CD31 in tumour sections demonstrated that tumours from ephrin-B2-sense-transfected KM12L4 cells (overexpressing ephrin-B2) had significantly higher tumour vessel counts compared to controls (*P*<0.0001, Figure 3A

). Immunohistochemical staining for PCNA to determine tumour cell proliferation rates revealed that ephrin-B2 overexpression led to significantly lower numbers of PCNA-positive cells than in controls (*P*<0.0001) (Figure 4

). However, careful inspection of the vessel morphology demonstrated small, thin-walled vessels (Figure 5

). Representative results of immunostaining are shown in Figure 5.

### Effect of ephrin-B2 overexpression on functional blood volume/flow in tumours

Overexpression of ephrin-B2 led to an increase in tumour angiogenesis (vessel count by CD31 staining) but a decrease in tumour size and tumour cell proliferation (PCNA staining). This apparent paradox could be explained by the development of an inefficient blood supply in the ephrin-B2-overexpressing tumours. Therefore, we hypothesised that despite more vessels being present in the ephrin-B2-overexpressing tumours, these tumours have a decreased functional vascular volume/flow due to inefficient blood supply. To investigate this hypothesis, we used ^51^Cr-labelled RBCs to determine blood volume in the tumours. ^51^Cr-labelled RBCs were injected intravenously via the tail vein into tumour-bearing mice (pcDNA3 and ephrin-B2 S2 transfectant, 10 mice/each group). Tumours from ephrin-B2-sense-transfected cells had a significantly lower blood volume per gram of tissue than did tumours from pcDNA3-transfected cells (*P*=0.0288) (Figure 3B).

## DISCUSSION

Ephrins consist of two classes: ephrin-As, which bind to the membrane via a glycosylphosphatidylinositol anchor, and ephrin-Bs, which interact with their receptors through the transmembrane cytoplasmic domain. Ephrins/Ephs are essential mediators in the development of the nervous (Flanagan and Vanderhaeghen, 1998) and cardiovascular systems (Wang *et al*, 1998).

Among the ephrin family members, ephrin-B2 is critically important for embryologic vascular development. Ephrin-B2 knockout mice resulted in severe vascular developmental defects during embryogenesis (Wang *et al*, 1998; Adams *et al*, 1999). In murine models, ephrin-B2 is expressed in the arterial ECs but not in the venous ECs (Wang *et al*, 1998; Adams *et al*, 1999). Ephrin-B2 is also expressed in the vascular smooth muscle cells of arteries (Shin *et al*, 2001). Wang *et al* (1998) reported that ephrin-B2s on arterial ECs interact with EphB4s on venous ECs, and Zhang *et al* (2001) showed that stromal cells expressing ephrin-B2 are involved in vascular network formation and proliferation of ECs. These data suggest that the interaction of ephrin-B2 and EphB4 leads to reciprocal signalling between arterial ECs, venous ECs, and surrounding vascular supportive cells (Wang *et al*, 1998; Zhang *et al*, 2001).

Further studies suggest that ephrin-B2 is involved in tumour angiogenesis. Shin *et al* (2001) observed ephrin-B2-expressing ECs within the vasculature of tumours, and ephrin-Bs and Ephs have been found to be expressed by a variety of human solid tumours and human tumour cell lines (Vogt *et al*, 1998; Tang *et al*, 1999a, 2000; Takai *et al*, 2001; Liu *et al*, 2002; Varelias *et al*, 2002). Overexpression of ephrin-Bs/Ephs is associated with increased tumour growth, increased tumorigenicity, and poorer prognosis in human osteosarcoma, endometrial cancer, and melanomas (Vogt *et al*, 1998; Takai *et al*, 2001; Varelias *et al*, 2002). Vogt *et al* (1998) reported that ephrin-B2 mRNA expression is higher in metastatic melanoma cell lines than in isogenic primary melanoma cell lines and higher in metastatic human tumours compared to primary melanomas. Vogt *et al* also showed a significant increase in ephrin-B2 mRNA expression in primary melanoma cell lines selected for increased tumorigenicity.

We transfected the full-length ephrin-B2 cDNA into the human colon cancer cell line KM12L4 and found that the *in vivo* growth of ephrin-B2-overexpressing tumours was significantly decreased compared to control tumours. Immunohistochemical staining showed that the tumours overexpressing ephrin-B2 had increased vessel counts but decreased tumour cell proliferation. Theoretically, decreases in tumour growth can be attributed to several broad categories of processes: (1) increased tumour cell death, (2) decreased tumour cell proliferation, and (3) alterations in angiogenesis that can affect both processes. Using Western blot analysis, we determined the protein expression profiles for regulators of the cell cycle (cyclins A, D1, D3, and E; cyclin-dependent kinases 2 and 4; and cyclin-dependent kinase inhibitors p21 and p27) and of apoptosis (the oncogene *murine double minute 2*; tumour suppressor genes *pRB* and *p53*; and apoptosis-related genes *caspases 2, 3*, and *7*) in ephrin-B2-transfected and control cells. These studies were carried out to determine whether ephrin-B2 transfection altered any of these parameters that could directly affect tumour growth *in vivo*. None of these proteins was altered by ephrin-B2 transfection (data not shown). Furthermore, cell cycle analysis by fluorescence-activated cell sorting showed that the cell cycle distribution of ephrin-B2 transfectants was the same as that of control cells (data not shown). In addition, an *in vitro* analysis of cell proliferation/number (MTT assay) likewise did not show any differences among groups (data not shown). Therefore, based on these analyses, we concluded that the decrease in tumour growth observed in the current study was not due to changes in the cell cycle or tumour cell apoptosis. We therefore hypothesised that despite the increase in microvessel density, these vessels were dysfunctional. To evaluate the ‘functional’ tumour vascular volume, we injected ^51^Cr-labelled RBCs and harvested blood and tumours to obtain the relative radioactivity. Tumours from ephrin-B2-transfected cells had a significantly smaller tumour blood volume despite having an increase in microvessel density. These data demonstrated that overexpression of ephrin-B2 can increase tumour microvessel density but that these vessels may be ‘dysfunctional’. The fact that these tumour vessels were dysfunctional is similar to the findings of Jain and co-workers (Jain, 2001). Our observation suggests that vessel density, although typically associated with angiogenesis, does not always reflect the functional status of the vascular network in tumours. Functional studies such as noninvasive imaging techniques may more accurately reflect the true functional status of the tumour microvascular bed than do anatomic or morphologic studies.

In summary, our study demonstrated two novel findings with potentially important implications. The first was that overexpression of ephrin-B2 on tumour cells can markedly decrease the growth of SQ colon cancer xenografts. The second interesting finding was that this decrease in tumour growth occurred despite an increase in tumour vessel counts. Our experiment with ^51^Cr-labelled RBCs suggested that tumour vessel count does not always correlate with the ‘functional status’ of the vasculature. This further questions the value of vessel counts as a measure of angiogenic or antiantiangiogenic activity (Hlatky *et al*, 2002) and suggests that functional studies more accurately measuring blood flow may be a better indicator of the functional tumour vascular network than morphologic or anatomic studies.
